# Melanotic neuroectodermal tumor of infancy in an African-indigenous patient from the Amazon: a case report

**DOI:** 10.1186/1746-160X-9-35

**Published:** 2013-11-25

**Authors:** Thiago Pastor da Silva Pinheiro, José Thiers Carneiro, Sérgio de Melo Alves, João de Jesus Viana Pinheiro, Fabrício Mesquita Tuji

**Affiliations:** 1 Oral radiology department, Dentistry posgraduation program, Federal University of Pará, Belém, PA, Brazil; 2 Ophyr Loyola Hospital, Belém, PA, Brazil; 3 Dentistry posgraduation program, Federal University of Pará, Belém, PA, Brazil; 4 Oral Pathology department, Dentistry posgraduation program, Federal University of Pará, Belém, PA, Brazil

**Keywords:** Infant, Melanotic neuroectodermal tumor of infancy, Progonoma, Imaging features

## Abstract

Melanotic neuroectodermal tumor of infancy (MNTI) is a rare condition that occurs normally in the anterior maxilla of infants aged <1 year. The use of clinical and imaging tools for MNTI is extremely important to prevent problems with function as well as the aesthetic features in a breastfeeding child. Brazil is a multiethnic country with a poor epidemiological policy and little data to track the incidence of certain diseases, including MNTI. It is important to study this pathology with ethnicity as a factor to improve the current epidemiological programs and establish better post-treatment management. This paper describes a case of a 2-month-old male of African-indigenous descent and Brazilian Amazon residency, who presented to our unit in 2009 with a history of an expanding mass involving the anterior maxilla. Clinical and computerized tomography scans were used to diagnose the mass as MNTI, which was removed by total excision. A biopsy later confirmed the MNTI diagnosis.

## Background

Melanotic neuroectodermal tumor of infancy (MNTI) is an extremely unusual, benign osteolytic neoplasm of neural crest origin [1] previously referred to as melanoameloblastoma, pigmented congenital epulis, melanotic progonoma, and other synonymous terms [2], and is classified as a congenital anomaly of the face and neck according to WHO ICD-10 [3].

MNTI affects infants in the first year of life [4] with no sex predilection [5], and since the time it was first reported by Krompecher in 1918 [6], approximately 360 cases have been described in the medical literature. Of these, Rustagi, Roychouldbury, and Karak found 237 cases (65.5%) involving the maxilla alone [7] (Table 1).

**Table 1:** MNTI involving the maxilla as reported in the literature

**Range of year**	**Total number of cases**	**Mean age (month)**	**Male: female ratio**
1918–1979	93	3.9 ± 1.4	0.95
1980–1989	37	3.6 ± 0.4	0.80
1990–1999	58	4.4 ± 0.3	0.80
2000–2009	49	4.1 ± 1.5	2.12
2010–2011*	11	3.5 ± 3.25	0.66
**Total**	**248**	**3.9 ± 2.6**	**1.06**

*Present case included.

Original data until 2009 from Rustagi, Roychouldbury, and Karak, 2011.

Within the data described in Table 1 between 2000 and 2011, there are no reports of MNTI in patients of African-indigenous descent or Brazilian Amazon residence.

This case reports MNTI in a 2-month-old male of African-indigenous descent, who presented to the oral surgery department at Ophir Loyola Hospital in Belem, Para, Brazil, and is to our knowledge the first such case reported in the literature.

### Case presentation

A 2-month-old normally developed male of African-indigenous descent (a background in Brazil referred to as “cafuzo”) with no relevant medical history presented to the oral surgery department at Ophir Loyola Hospital (Belem, Para, Brazil) in 2009 with a history of an expanding mass that involved the anterior maxilla, observed since his birth, that interfered with breathing and feeding.

**Figure 1:**
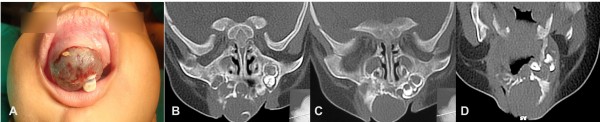
**Prematurely erupted teeth with a firm, non-ulcerated, reddish-bluish tumor approximately 4 cm × 4 cm in size, extending from the alveolar ridge to the hard palate and covered by intact mucosa. A**, **B**, **C**, **D**: Pre-operative axial computed tomography scans showing an erosive and expansive lesion extending from the alveolar ridge to the hard palate and regional displacement of teeth.

Oral examination showed prematurely erupted teeth and a firm, non-ulcerated, reddish-bluish tumor of approximately 4 cm × 4 cm in size, extending from the alveolar ridge to the hard palate, displacing the overlying cheek and lip and covered by intact mucosa (Figure 1).

Computed tomography (CT) in the soft tissue window showed an expansive mass measuring 4 × 4 × 3 cm that involved the middle of the anterior maxilla region with bone destruction, extending superiorly and medially, just inferior to the ethmoid air cells. Several unerupted tooth buds were displaced laterally (Figure 1 A, B, C, D).

**Figure 2:**
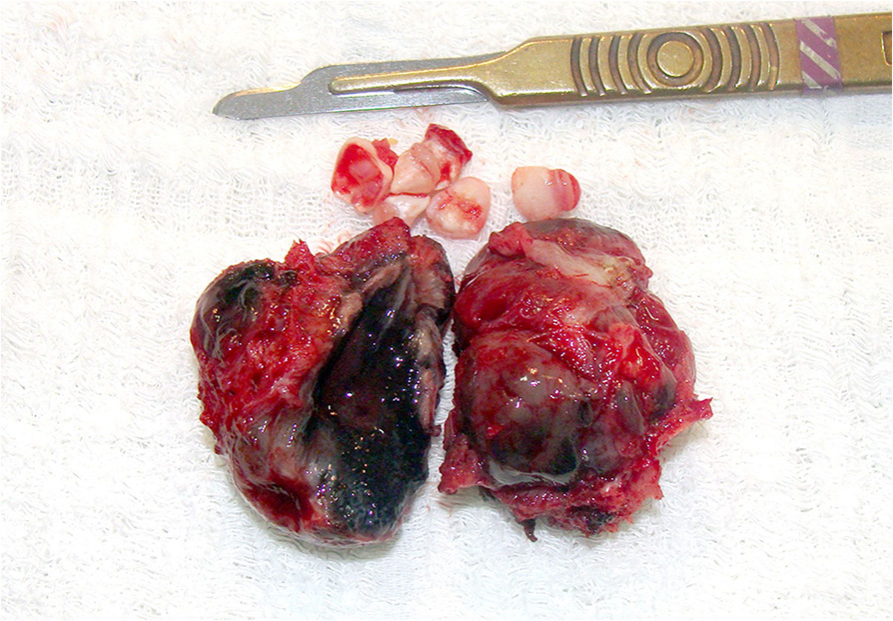
Tumor mass was totally excised in two pieces, with developing deciduous teeth embedded within the mass.

**Figure 3:**
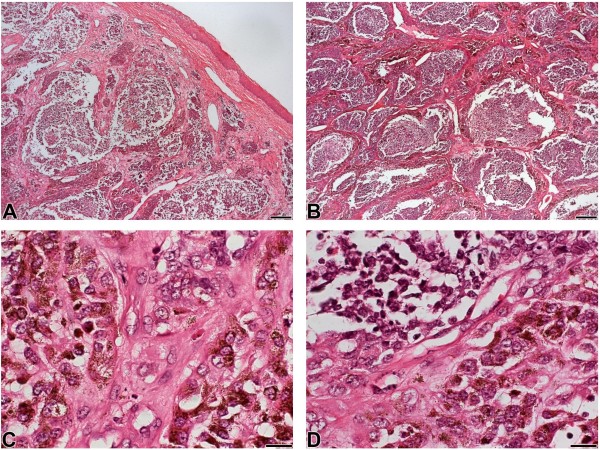
**A, B: Postsurgical facial appearance. A**, **B**: Note the alveolar pattern and the fibrous stroma (hematoxylin and eosin, scale bars 200 μm); **C**: Larger cells with intracellular melanin granules (Hematoxylin and eosin, scale bar 20 μm); **D**: The biphasic microscopic pattern (Hematoxylin and eosin, scale bars 20 μm).

The chosen treatment was enucleation. An incision was made under general anesthesia over the covering mucosa, followed by displacement, divulsion, and curettage, exposing the tissue injury. The lesion was large and well bonded to the maxilla, permitting a two-piece excision of the mass. Dental germs were observed mixed within the lesion (Figure 2). Immediately after removal of the lesion, the patient’s facial appearance improved and the alveolar ridge showed some degree of normality (Figure 3).

A microscopic biopsy later showed that the tumor was composed of two different types of cells. One portion of the lesion presented smaller round cells with minimal cytoplasm and hyperchromatic nuclei, whereas the other portion exhibited larger cells with vesicular nuclei and eosinophilic cytoplasm containing typically abundant brown intracellular melanin granules. The first cell population (neuroblast-like cells) was arranged in nests or in alveolar patterns surrounded by the larger ectodermal cells, separated by fibrovascular stroma; this biphasic pattern characterized MNTI (Figure 3).

The patient presented with excellent clinical features and normal feeding and breathing functions at the 1-week, 2-week, and 1-month follow-up examinations. The facial growth has been normal thus far, but long-term oral rehabilitation will be necessary because of teeth extraction. No recurrence was detected.

## Discussion

This case involved a patient who presented with a classical description of MNTI, showing a lesion involving the maxilla, which affects 68–80% of children in the first year of life. It has also been reported to involve the mandible (5.8%) and other sites with minimal incidence, such as the cerebellum, brain, femur, epididymis, pineal region, skull, mediastinum, shoulder, oropharynx, thigh, uterus, ovary [5], and frontotemporal sphenoid areas [8].

The lesions are usually benign, presenting as an aggressive, quick growing, and painless mass in the anterior maxilla [9] with irregular reddish-bluish pigmentation swelling on the top of the alveolar crest [10]. This mass may have a rubbery consistency and non-ulcerated surface, containing prematurely erupted or displaced primary teeth [11].

Generally, these tumors are asymptomatic lesions, noticed by parents because of asymmetry caused by a growing mass in the midface region [12] that displaces teeth and deforms the patient’s face. In our reported case, the patient’s mother had noted the lesion since birth, and it was treated 2 months later, affecting function as well as the facial features in the breastfeeding child. Normal facial growth was observed at the first post-surgical follow-up examination, but the child had 5 teeth missing up to the age of 10 years. This condition could lead to facial development problems; therefore, frequent dental follow-up examinations are required, with oral rehabilitation treatment.

Despite the classic features mentioned, this patient was of “cafuzo” descent living in the Brazilian Amazon. Among all the cases described in Table 1 between 2000–2011, this is the only report of MNTI in a patient with this specific ethnic background and residence. Previous studies have shown a variety of ethnicity-related dental conditions and disorders such as cleft lip and palate [13], a varied amount of cancer subtypes [14], and some fibro-osseous lesions such as florid osseous dysplasia, focal cemento-osseous dysplasia, and periapical cemental dysplasia [15].

We suggest that the behavior of MNTI in “cafuzo” patients is similar to that in individuals of other races, showing the influence of familial ethnicity (a mix of African and the indigenous Brazilian population), for which further research is needed.

Radiographs and CT images have revealed the following characteristics of MNTI: tissue destruction, poorly demarcated radiolucency, hypodense underlying bone, occasional faint “sunburst” appearance resulting from mild calcifications along vessels radiating from the center of the tumor [11,12]. The imaging results, and laboratory and clinical examinations are essential in preoperative diagnosis. Magnetic resonance imaging and vanillylmandelic acid (VMA) tests were not performed in this patient; however, CT scans were used to characterize the lesion, identify dental abnormalities, and delineate the extent of the tumor to aid resection and to anticipate the reconstruction of the above-mentioned functions and oral rehabilitation needs [16].

The surgical approach for lesion removal was enucleation. This conservative surgical approach consists of local excision and curettage, and prevents aesthetic sequelae with reduced manipulation of the lesion. While extent of surgical excision is debatable in the literature [4], the chosen surgical method seems essential to reduce the possibility of local recurrence rates to approximately 10–15% [17,18] and usually leads to a good prognosis [19].

In spite of the classical clinical and imaging features presented in our case report, only a histopathological evaluation could confirm MNTI [2], demonstrating the necessity of this diagnostic protocol for this disease.

## Conclusion

Brazil is a multiethnic country with a poor epidemiological policy and little data to track the incidence of certain diseases, including MNTI. Studying this pathology while considering ethnicity is important to improve current epidemiological programs. The low number of MNTI cases reported in “cafuzo” patients and in the residents of the Brazilian Amazon region demonstrates the need to register such cases and improve the local dentists’ knowledge about the classical features of MNTI to facilitate a preliminary diagnosis. Careful management of post-surgical consequences such as the loss of teeth and bone support is needed in patients of mixed ethnic backgrounds such as our patient. When designing facial orthopedics to help with prosthetic rehabilitation and/or reconstructive surgery in adults, the integrity of the facial pattern in the region should be preserved, providing an aesthetic face.

### Consent section

Written informed consent was obtained from the patient for publication of this Case report and any accompanying images. A copy of the written consent is available for review by the Editor-in-Chief of this journal.

## Competing interests

The authors declare that they have no competing interests.

## Authors’ contribution

TPS followed the patient since the diagnosis, obtained photographs, reviewed the literature, and wrote the manuscript. JTC performed the surgery and followed up with the patient. SM and JJV performed the histopathological diagnoses and provided histopathological slide photographs. FM developed the study concept, participated in its design and coordination, and helped to draft the manuscript. All authors read and approved the final manuscript.
